# Constraints on natural global atmospheric CO_2_ fluxes from 1860 to 2010 using a simplified explicit forward model

**DOI:** 10.1038/srep17352

**Published:** 2015-11-27

**Authors:** Helge Hellevang, Per Aagaard

**Affiliations:** 1 Department of Geosciences, University of Oslo, P.O. Box 1047, Blindern, NO0316 Oslo, Norway; 2 The University Centre in Svalbard (UNIS), Pb. 156, 9171 Longyearbyen, Norway

## Abstract

Land-use changes until the beginning of the 20^th^ century made the terrestrial biosphere a net source of atmospheric carbon. Later, burning of fossil fuel surpassed land use changes as the major anthropogenic source of carbon. The terrestrial biosphere is at present suggested to be a carbon sink, but the distribution of excess anthropogenic carbon to the ocean and biosphere sinks is highly uncertain. Our modeling suggest that land-use changes can be tracked quite well by the carbon isotopes until mid-20^th^ century, whereas burning of fossil fuel dominates the present-day observed changes in the isotope signature. The modeling indicates that the global carbon isotope fractionation has not changed significantly during the last 150 years. Furthermore, increased uptake of carbon by the ocean and increasing temperatures does not yet appear to have resulted in increasing the global gross ocean-to-atmosphere carbon fluxes. This may however change in the future when the excess carbon will emerge in the ocean upwelling zones, possibly reducing the net-uptake of carbon compared to the present-day ocean.

It is beyond doubt that anthropogenic emissions (from burning of fossil fuel, energy intensive industry and land-use changes) are the cause of the exponentially increasing atmosphere CO_2_ as observed since the onset of the industrial revolution^1^,^2^,^3^,^4^,^5^. The increasing atmospheric CO_2_ concentration is a key factor for climate changes, and this has placed knowledge about the global carbon cycle in the forefront of policy debates and climate research. To implement effective carbon-related policies and to develop future carbon emission trading, a good understanding is required of the carbon sinks and sources and the human impacts on them.

The relative contribution of the emissions and the efficiency of the biosphere and the ocean to mitigate the increase in atmospheric CO_2_-concentrations, remain highly uncertain^2^,^5^,^6^,^7^,^8^. This is demonstrated in chapter six of the latest IPCC report^5^, where we can read that the net land-atmosphere carbon flux in the 1980s was estimated to −0.1 ± 0.8 Gt C/a (negative numbers denote net uptake). These numbers were partly based on estimates of net CO_2_ releases caused by land use changes (+1.4 ± 0.8 Gt C/a), and a residual terrestrial sink estimated to −1.5 ± 1.1 Gt C/a. There are globally much data supporting increased uptake of carbon by the ocean mixed layer (shallow surface water)^9^,^10^,^11^,^12^,^13^ but the global gross ocean-atmosphere fluxes, partly influenced by annual and inter-annual processes, such as El Niño/La Niña events^14^,^15^, are nevertheless not easy to estimate. Obtaining global values of the carbon fluxes are further complicated by large local and regional variations in carbon releases and uptake by the terrestrial biosphere^5^,^8^,^16^,^17^. Because of the close coupling between oxygen and carbon fluxes during photosynthesis and respiration, the tracer APO (Atmospheric Potential Oxygen), in combination with atmospheric CO_2_ data, is used to obtain the net amount of CO_2_ being taken up by the oceanic sink^16^. The net amount of carbon being taken up by the terrestrial biosphere can then be found from the residual (difference between carbon accumulated in the atmosphere and amount taken up by the global oceans)^16^,^18^. APO values are however not straightforward to estimate, and a recent study suggests that the strength of the terrestrial sink may be significantly lower than found earlier^19^. Moreover, current measurements of the atmospheric O_2_/N_2_ ratio and CO_2_ concentrations may suggest that the amount of oxygen is dropping at a faster rate than calculated from the APO tracer values^20^.

Biomass burning and conversion from forest to agricultural land, contribute at present approximately 10% of the total anthropogenic emissions^5^,^21^. Such land-use changes are driven by the increasing demand for fertile farm land, leading to large scale soil degradation^22^,^23^,^24^,^25^. In the 18^th^ and 19^th^ centuries and until the first half of the 20^th^ century, rapid expansions of farming and deforestation were the main sources of anthropogenic CO_2_ to the atmosphere^26^,^27^. Deforestation and soil degradation are however still ongoing at alarming rates^28^. At present, burning of fossil fuel and cement production contribute about 10 Gt/a of carbon emissions, whereas land-use changes are estimated to provide about 0.9 Gt C/a^5^. The stable carbon isotopes (^12^C and ^13^C) and their isotope ratio (*R* = ^*13*^*C/*^*12*^*C*) reflect the processes distributing carbon between the various reservoirs (photosynthesis, respiration, ocean dissolution etc.) and the related isotope fractionation. For convenience, values are compared to a standard value and the *δ*^*13*^*C* notation, also referred to as the “carbon isotope signature” is used:




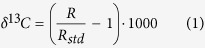




The value of the standard ratio *R*_*std*_ is by convention that of the Vienna Pee Dee Belemnite (VPDB)^29^. In the terrestrial biosphere, photosynthesis preferentially takes up the light carbon isotope (^12^C) in plants, and the terrestrial biosphere therefore has a negative *δ*^*13*^*C* value, on average −25.0%^13^. Photosynthesis in the surface ocean water removes the light carbon isotopes from the water, giving the water a slightly positive *δ*^*13*^*C* value (at present about +1 to +2%)^11^.

The carbon isotope signature (*δ*^*13*^*C*) of the atmosphere is affected by carbon exchanges with the biosphere and ocean mixed layer, and from anthropogenic carbon emissions. Because of the changes in these fluxes with time, and most importantly anthropogenic contributions to the fluxes, the atmospheric *δ*^*13*^*C* has changed significantly since the 19^th^ century, from a value of about −6.5 to a present-day value below −8.0 (Fig. 1)^30^,^31^,^32^,^33^. This is caused by the depletion of atmospheric ^13^C by the addition of ^12^C-enriched carbon from fossil fuel and land-use changes (the so called Suess effect). The rate of change has increased considerably during the 20^th^ century, presumably due to the accelerating input from fossil fuel burning which releases carbon with a strongly negative *δ*^*13*^*C* (at present about −28)^34^.

In this study, we propose a new simple box model solved in forward mode to evaluate the sensitivity of the atmospheric carbon isotope signature on natural and anthropogenically induced carbon fluxes (see *Methods*). Natural here refers to atmosphere-biosphere and atmosphere-ocean carbon fluxes prior to significant input from land-use changes (pre-19^th^ century fluxes). A forward model is a numerical algorithm where fossil fuel emissions, carbon-emissions from land-use changes, base (at time zero) natural fluxes, isotope fractionation, and partitioning of excess carbon is used as input, and the carbon isotope signature of the atmosphere is calculated. The aim is to extend earlier forward models including input data of the atmospheric carbon isotope inventory, land-use changes, fossil-fuel emissions, and isotope signatures of fossil fuel to 2010. Some datasets, e.g., ice core data from the Law Dome and South Pole, have recently been modified and extended^33^ and is compared to the modeling results. One main aim is to tune the global natural carbon fluxes, but the forward model also allows us to see if the main human sources of carbon emissions; fossil fuel burning and land-use changes, can be observed in the recent data of the atmosphere carbon isotopes. The forward modeling approach can furthermore be used to explicitly test to what extent some of the natural fluxes (e.g., the ocean-atmosphere carbon flux) is changing, or if the isotope fractionation factors can be regarded as constant with time. The model (eq. 2) used in this work is essential the same as the forward model used by Tans *et al.*^35^ and the derivation of the model can be found there. The model is simplified, and does not take into account isotope mixing between shallow and deep ocean waters, but instead calculate the shallow-water carbon isotope inventory as a linear function of changes in the atmosphere inventory (eq. 6 provided in *Methods*). This reduces the number of uncertain input parameters, but preserves the full capability of the model to predict changes in the atmosphere carbon isotope inventory. The isotope fractionation factors were also simplified from three to two parameters here, but this does not affect the modeling (both here and in Tans *et al.*^35^ constant fractionation factors were used for the entire simulated time span, and this is suggested to be valid from the comparison between modeling results and measured data). The way the model was solved also differed slightly. Tans *et al.*^35^ solved the model explicitly for the fluxes given the isotope disequilibria as input, whereas we varied carbon cycle parameters (fluxes, ocean/terrestrial biosphere sink/source strengths, etc.) and compared the model fit to the measured atmospheric carbon inventory. Finally, Tans *et al.*^35^ model was used successfully for a narrow time interval between 1970 and 1990, whereas this work has expanded the timeframe to 1860 to 2010. Other models used to constrain global carbon fluxes, are based on the Tans *et al.*^35^ equations, but run in inverse mode^33^,^36^,^37^ (e.g., using measured atmospheric *δ*^*13*^*C* and CO_2_ pressures to estimate fluxes) rather than in forward mode as used in this work (Trudinger *et al.*^37^ uses forward calculations but they are only implicitly given in the inverse model results). The inverse models have a much larger number of input parameters, and smoothening of the input *δ*^*13*^*C* and CO_2_ pressures is required. Some further discussion on challenges using the results from the inverse models is provided at the end of the results section.

## Results

In order to use the forward model to constrain parameters of the global carbon cycle, such as the major natural carbon fluxes, the airborne fraction (amount of anthropogenic CO_2_ that accumulate in the atmosphere), and amount of excess carbon being stored in the ocean and terrestrial biosphere, we performed a sensitivity study where we changed parameters one by one (while fixing the other parameters) and observed the effect on the modelled carbon isotope signatures. Table 1 summarizes the base-case parameters used, and the background for these values is explained in the texts below.

### Base-case parameterization

See table 1 for an overview of variables used in the model, including their base-case and/or initial values and their literature sources. The remaining of this section is devoted to explain the background for the chosen values. Further details on the model equations with explanations are given in the *Method* section.

**Figure 2:**
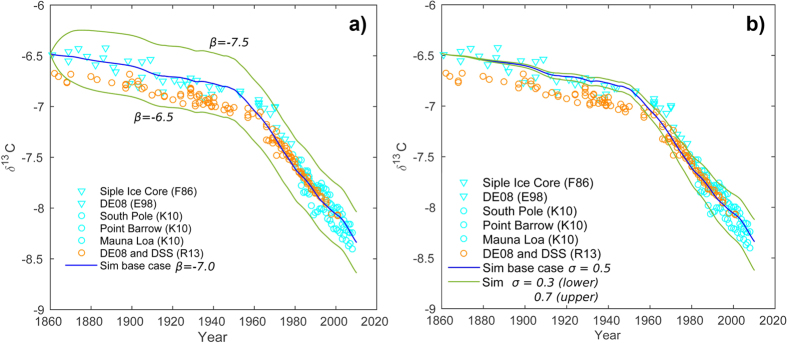
(**a**) Optimizing the *β* parameter (correcting for isotope fractionation upon photosynthesis) to provide initial steady-state atmospheric 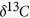 values. The *β* value depends on the choice of terrestrial (*J*^*(ba)*^) and ocean (*J*^*(oa)*^) carbon fluxes to the atmosphere, and a value of −7.0% was found for the base case (*J*^*(ba)*^ = 60 Gt C/a, *J*^*(oa)*^ = 78 Gt C/a and *α* = 0.0%). (**b**) Sensitivity of the model to *σ*, providing the change in surface ocean δ^13^C as a fraction of the change in the atmosphere δ^13^C (Eq. 5).

To obtain a set of parameters to use for the base case, we used initial ocean-atmosphere (*J*^*(oa)*^) and biosphere-atmosphere (*J*^*(ba)*^) fluxes of 78.0 and 60.0 Gt/a respectively. The remaining input parameter, the isotope fractionation factor *ε*_*b*_ (with *ε*_*o*_ = *0*), was found by the requirement of a steady-state atmospheric isotope signature (shortened 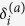) for the initial time after 1860. With this base-case setup, we found that a value for *ε*_*b*_ of −7.0% provided the best fit. Simulated 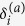 values are generally within the range of measurements for the entire simulated time, except for the period from 1962 to 1978 where the modelled 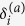
is slightly lower than the Law Dome DE08 ice core data set (Fig. 2a). In our comparison however, the DE08 ice core provides the only time series for this period, and we would expect also a range of 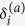 values here if we take into account regional variations comparable to the data recorded during the later years (approximately ±0.15%). A variation in *ε*_*b*_ of ±0.5% illustrates the sensitivity of the model to this parameter (Fig. 2a). Notice how the three curves are close to parallel after initial steady-states have been reached, and also parallel to the measured data recording the accelerated changes that have pertained since the middle of the 20th century.

The change in surface ocean *δ*^*13*^*C* is modelled as a fraction of the change in 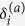, and therefore adopts the same slopes. With the starting δ^13^C value of +2.5 and a *σ* (see eq. 6) of 0.5, we obtain a present-day value of about +1.55, which is in good agreement with recent observations (Fig. 2b)^9^,^10^,^12^. It is interesting to see that, due to the relatively large seawater-atmosphere carbon fluxes, even modest changes in ocean *δ*^*13*^*C* have a significant impact on 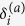 (Fig. 2b).

**Figure 3:**
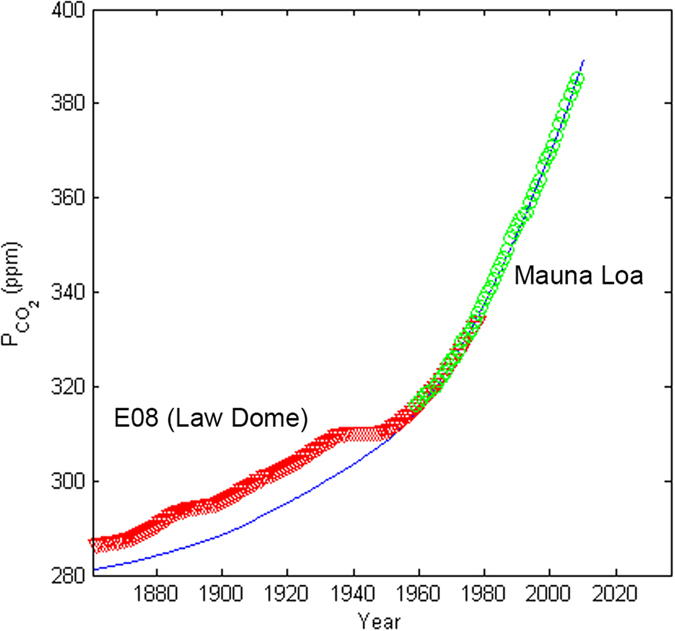
Comparison of simulated and measured atmospheric CO_2_ partial pressures. The measured data are from the Law Dome DE08 ice core (red triangles)^31^, and from the Mauna Loa sampling station (green circles)^32^ respectively.

The simulated changes in CO_2_ pressure and a comparison with data by Etheridge *et al.*^31^ based on Antarctic ice-core measurements (Law Dome, DE08), and recent direct measurements reported by Keeling *et al.*^32^ is shown in Fig. 3. The comparison suggests that the anthropogenic fluxes and the assumption of 46% airborne CO_2_ provide good estimates for the changes in atmospheric CO_2_ mass and corresponding CO_2_ pressure. The significant difference between simulated and measured data in the early part of the comparison (1860 to 1950) is however not understood. Some studies have suggested higher values for the fraction of anthropogenic carbon accumulating in the atmosphere. For example, Rafelski *et al.*^38^ assumed an airborne fraction of 57%, which also gave good predictions of the CO_2_ pressures. They, however, did not include excess fluxes from land-use changes, as was done in the present study.

### Sensitivity of model to the terrestrial biosphere-atmosphere carbon flux

**Figure 4:**
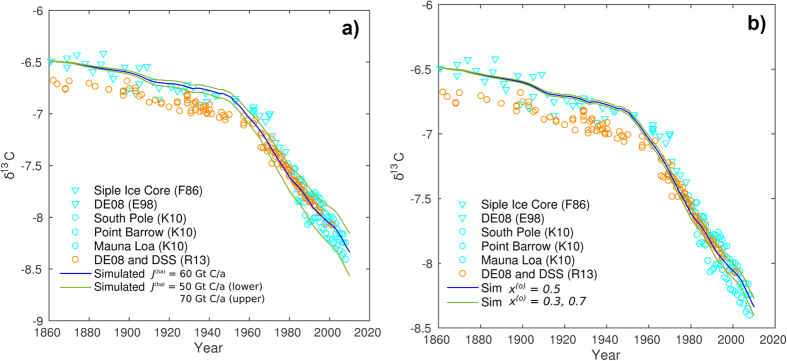
(**a**) Sensitivity of the model to the non-anhtropogenic part of the biosphere-atmosphere flux (*J*^*(ba)*^). The simulations were done with a fixed value of *x*^(*o*)^ = 0.5, implying that the ocean takes up 50% of the residual carbon being stored in the terrestrial biosphere-ocean system. (**b**) Sensitivity of the model to the fraction of excess carbon being stored in the ocean relative to the terrestrial biosphere. The simulations were done with a fixed non-anthropogenic biosphere-atmosphere flux of 60 Gt C/a.

In our model carbon fluxes between the terrestrial biosphere and atmosphere were divided into two distinct parts; one large component representing fluxes prior to human influences by land-use changes and fossil-fuel burning (hereafter referred to as the base terrestrial-atmosphere carbon flux), and one smaller but increasing component including the contributions from human perturbations of the system (including the increasing net uptake of carbon by land-plants caused by increasing atmospheric CO_2_). The base terrestrial-atmosphere carbon fluxes were held constant from 1860 to 2010. We first attempted using fluxes of 60 Gt C/a, in accordance with the use of a net primary production (NPP) of about half the gross primary production (GPP)^39^, and with GPP typical estimated to 120 Gt C/a^5^,^39^. Exact values of gross fluxes are hard to estimate, and they are generally quoted with uncertainties greater than ±20%^5^. Simulations using base terrestrial-atmosphere fluxes of 50 and 70 Gt C/a were therefore also performed, roughly covering the entire range in uncertainty. This range also covers the somewhat lower estimates by Ito^40^ and Runnin^41^, suggesting NPP of 56 and 54 Gt C/a respectively. The results are shown in Fig. 4a. The modeling shows that the difference between the models is small prior to about 1960. From thereon, however, using a base flux of 50 Gt C/a apparently overestimates the rate of reduction of the atmosphere δ^13^C, giving estimates on the lower side of the data assemblage, whereas the opposite is true using 70 Gt C/a. The base-case value of 60 Gt C/a gives the overall best fit to the measured data points, further supporting the use of such a value in the global carbon cycle models.

Despite the general good fit between the simulated and measured data, there is apparently a mismatch between the updated ice core data from Rubino *et al.*^33^, and the recent firn data (Fig. 4a). The fit between the model and measured recent data is very good, but it is impossible to obtain this fit with a *δ*^*13*^*C* starting point (1860) of about −6.65%, required for the shifted ice-core data. Instead, using the old un-shifted ice-core data (*δ*^*13*^*C* starting point (1860) of about −6.48%) provides excellent fit between model and measured data for the entire period from 1860 to present. As will be discussed further below, this has consequences for the inverse modeling and estimated natural fluxes^33^, especially for transition period from ice- to firn data.

### Relative contributions of the ocean and terrestrial biosphere sources/sinks

**Figure 5:**
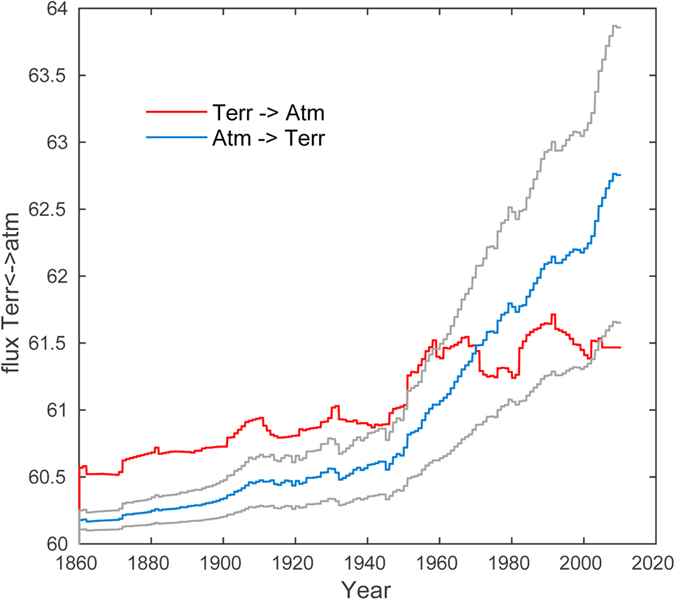
Carbon fluxes from the biosphere to the atmosphere (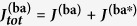) (red curve) compared to the simulated counter-fluxes from the atmosphere to the biosphere (*J*^*(ab)*^). Three scenarios are simulated varying the fraction of excess carbon being taken up by the ocean (*x*^(*o*)^): the base-case scenario with *x*^*(o)*^ = 0.5 (blue curve); one with lower oceanic carbon uptake (*x*^*(o)*^ = 0.3) (upper grey curve); and one with a higher uptake (*x*^*(o)*^= 0.7) (lower grey curve). The biosphere is a net carbon sink when 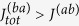.

In the model base-case we assumed that equal amounts of the excess atmospheric CO_2_ would be taken up by the ocean (through increased dissolution following Henry’s law) and the terrestrial biosphere (through increased plant growth) respectively, and we further assumed that this have not change significantly between 1860 and present. The true fraction of carbon taken up by the terrestrial biosphere is however lower, because of carbon emissions from land-use changes (treated as a separate input parameter in the model). The terrestrial biosphere has therefore for most of the periode 1860 to present been a net source of carbon. The total amount of excess carbon taken up by the two systems was estimated using an airborne fraction of 0.46, implying that a residual of 54% of the anthropogenic carbon emissions each year have been taken up by the two sinks. To understand how changes in the individual contributions of the two sinks affect the carbon isotope signature, we compared simulations using ocean uptake of 30, 50 and 70% of the residual 54% excess carbon. The sensitivity study suggest that modelled atmospheric *δ*^*13*^*C* is little sensitive to changes in the relative contributions of the ocean and biosphere to take up the anthropogenic CO_2_ (Fig. 4b). As already mentioned, values can be constrained by the APO tracer, and a range of studies suggests that the terrestrial biosphere has been a net sink since the second half of the 20th century, and that the ocean and biosphere sinks recently have made quite similar contributions^5^. The time when the biosphere became a net sink can also be found in the present modeling, by comparing the natural and anthropogenic biosphere emissions to the simulated counter flux. If we use the base-case values, the biosphere has been a net sink since 1970 and the net uptake at present (2010 value) is about 1.3 Gt C/a (Fig. 5) (compared to a present-day ocean uptake of about 3.6 Gt C/a). These values are in the range of earlier estimates^5^,^17^,^38^, but the timing is later and the size of the flux is on the lower end of earlier estimates. If the fraction of excess anthropogenic carbon being stored in the biosphere is changed to 70 and 30%, the time at which the biosphere becomes a net sink is shifted to the years 1950 and 2000, respectively. The corresponding net 2010 uptakes then change to 2.4 and 0.2 Gt C/a. The present-day net land surface uptake is estimated to approximately 1.4 to 3.8 Gt C/a (mean about 2.6–2.9 Gt C/a)^5^,^42^, indicating that the fertilization of land plants are more effective than ocean uptake in taking up excess atmospheric carbon, but keep in mind that much of this effect is lost by the human induced carbon emissions from land-use changes.

### Model constraints on temporal changes in the ocean-air CO_2_ flux

The ocean-air flux has been proposed to have increased from 60 to 78 Gt C/a since 1750 following the textbook analysis of Sarmiento and Gruber^5^,^43^. The background for the increase is a complex response of surface ocean temperature, CO_2_ solubility, and biogenic carbon uptake on increased atmospheric CO_2_. In the present model, isotope fractionation factors (*ε*_*o*_ and *ε*_*b*_, see Methods) were used to tune the model to provide a good match between simulated and measured values for the atmosphere carbon isotope signature at the onset of the simulations (1860). Our forward model suggests that using constant values for the estimated isotope fractionation factors result in a good match between measured and simulated data for the entire period from 1860 to 2010. Constant fractionation factors have also been used by other forward and inverse models^33^,^35^,^36^, supported by the general idea that the large dominance of C3 plants, dominating in the temperate forests, has not changed very much over the last 150 years. The increases in the atmospheric CO_2_ result in moderate increases also for the atmosphere-ocean and atmosphere-terrestrial fluxes. If we now use a large increase in the ocean-atmosphere carbon fluxes from 1860 to present, as suggested by Sarmiento and Gruber^43^, and still utilize fixed values for the isotope fractionation factors, the modelled atmospheric *δ*^*13*^*C* plots very far from the measured values. This indicate that only modest changes in the ocean-air fluxes have occurred over the last 150 years. The possibility that the isotope fractionation factors have changed significantly and with a magnitude that perfects balances the changes in the ocean-atmosphere flux, is very unlikely. The reason why the ocean-air flux is not very sensitive to increasing atmospheric CO_2_ levels over time-scales of less than 150 years, is that ocean upwelling of old waters to a large degree dictates the ocean-atmosphere carbon flux^14^, with the upwelling waters being on average much older than 150 years^44^. Increased ocean-atmosphere fluxes are therefore expected in the future, and this may lead to decreasing net carbon uptake and a corresponding increasing airborne fraction.

### Individual contributions of land-use changes and fossil fuel to changes in δ^13^C–1860 to 2010

**Figure 6:**
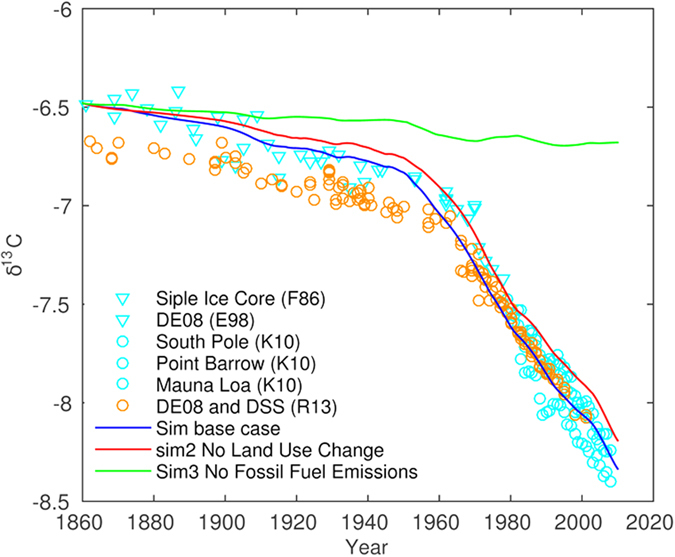
Individual contributions of anthropogenic emissions (land-use changes and fossil-fuel burning) to the modelled temporal changes in the atmospheric 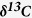. Both fluxes are needed to get good fits between measured values (and slopes) for the entire time frame, despite a decent fit if only the contribution from fossil-fuel burning is taken into account.

If we try to simulate the changes in the isotopes without taking into account any anthropogenic emissions, or with only fossil fuel burning or changes in land-use, we cannot obtain the slopes 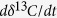 as observed in the measured data (Fig. 6). It is also indicated that fossil fuel burning alone is not sufficient to explain the slopes. Only the combined contributions of the two sources can fully explain the atmospheric *δ*^*13*^*C* over the simulated time from 1860 to 2010. There are also no other natural carbon sources with 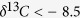 (about present day value) that can be imposed instead to get the slopes. Magmatic (mantle) degassing has a δ^13^C of about 0.0, and there are limits for how much the net carbon fluxes to the ocean can be increased. We also know that the terrestrial biosphere cannot have been a large net source of carbon over the last 50 years^5^. Claims by ‘climate sceptics’ that the build-up of carbon since the industrial revolution is mostly natural, using the argument that the residence time of carbon in the atmosphere is short and that little anthropogenic carbon is left, can therefore be easily refuted by studying the carbon isotope changes.

### Estimated natural fluxes: Forward vs inverse models

**Figure 7:**
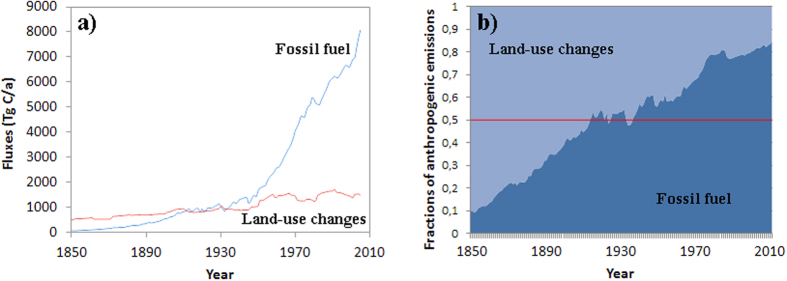
Carbon fluxes from fossil fuel burning from Boden *et al*.^47^ compared to fluxes from land-use changes from Houghton *et al*.^48^.

The present work is done using a forward model, largely based on the same equations as in Tans *et al.*^35^, but utilizing recent and updated data up to 2010 and also extending the simulations back to 1860. Tans work was shown to successfully model the effect of fossil fuel combustion on the atmospheric and ocean carbon isotope evolution, but did not include the contribution of land-use changes, estimated to contribute between ~20 and 40% of the total human carbon emissions during the time interval 1970–1990 (see Fig. 7). Hence, the natural and estimated induced carbon fluxes (especially the atmosphere-ocean fluxes) must have been shifted accordingly to ensure fit between model and measured isotope data.

Inverse models are based on single or double deconvolution algorithms, and with various extended methods (e.g., the Kalman Filter double deconvolution)^33^,^45^ to quantify uncertainties in estimated fluxes. These models have been utilized to estimate natural and induced carbon fluxes over much of the same time interval as in this study, 1860 to present, and the most recent one extended simulations to 2010^33^. The Kalman Filter double inversion methods are very good to quantify uncertainties, and much of our understanding of the uncertainties comes from these studies. The forward modeling approach offers no such quantitative method to assess uncertainties, but could potential be extended using statistical modeling such as adding a Monte Carlo algorithm, but this was outside the scope of this work. Despite the superiority in obtaining uncertainties, inverse models also have some clear limitations. First, de deconvolution methods require continuous data series, and uses smoothed curves fitted to the longer time series where data are few. Estimated fluxes will therefore, to some extent, depend on the choice of moving average. Second, the longer time series contains a mixture of data from different sources (e.g., measurements of ice air bubbles followed by direct measurements from atmospheric air), and these may not be well correlated. This implies that data (and the smoothed curves) may be shifted and this may have large impacts on the estimated fluxes. One example is the combination of ice core CO_2_ pressure data from the Law Dome (E08)^31^ and direct measurements at Hawaii^32^ (shown in Fig. 3) used in the inversion by Trudinger *et al.*^37^ Their model (and any other inversion using these data sets) would suggest that the flattening (actually slight drop) in CO_2_ pressures during the 1930s to 40s must be caused by a large increase in the net uptake of CO_2_ in the ocean. There is however no physical mechanism to explain this increased uptake during this time. Changes in the natural ocean carbon uptake is closely connected to strengthening and weakening of the downwelling/upwelling currents mixing deep and shallow ocean water masses, which is further connected to El Niño/La Niña events^14^,^15^. There are no indications of specific decrease in upwelling or increase in downwelling by particularly strong and frequent El Niño events during this time^46^. Also increased biogenic growth that would depend on a period of increased nutrient supply is not likely. Lacking any other plausible explanation for the increased carbon uptake during this time, the flattening of the data may just be an artifact. The inverse models do however not distinguishing between physical and unphysical background of trends in data and the estimated fluxes may therefore also be unphysical.

## Conclusions

The forward modeling approach offers an explicit way of testing how various parameters affect the isotope signature of the atmosphere. In this study we extended the work by Tans *et al.*^35^ in time. We found that the forward modeling approach, and even for the very simplified models, can be used to successfully model the temporal evolution of the atmospheric carbon isotope signature over extended times, here the 150 years between 1860 and 2010. This does however rely on input data on fossil fuel combustion, land-use changes, and the assumption that the isotope fractionation factors between the atmosphere and terrestrial biosphere and ocean are known (in this work successfully treated as constants). The modeling also suggests that outgassing of carbon from the ocean (mainly at ocean upwelling zones) has not increased significantly since 1860. This contrasts to the general consensus based on Sarmiento and Gruber^43^, and also used by IPCC^5^, that ocean-atmosphere carbon fluxes have increased from 60 to 78 Gt C/a since 1750. Finally, the simplified forward modeling approach used here does not offer the powerful capability of Kalman Filter Double Deconvolution to yield uncertainty estimates of fluxes^33^,^37^. On the other hand, the forward model is explicit and does not need any smoothening of data, and can be used as a complimentary method to constrain natural and induced global carbon fluxes.

## Methods

**Figure 8:**
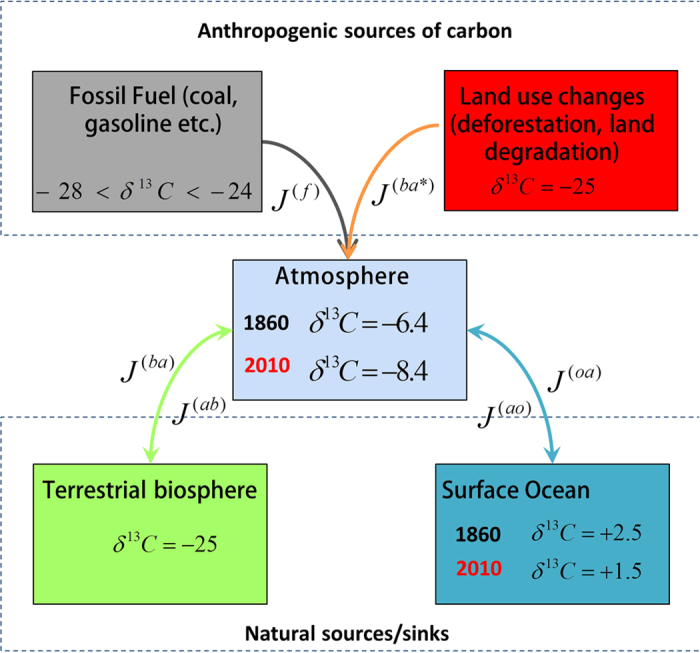
Box model representing atmospheric carbon stable isotope mixing by taking into account fossil fuel burning, and fluxes of carbon to and from the terrestrial biosphere and surface ocean water and the atmosphere.

The δ^13^C of the atmosphere was calculated for each year from 1860 to 2010 by using a three-box-model representing the terrestrial biosphere, ocean mixed layer, and atmosphere carbon reservoirs, and anthropogenic carbon fluxes from fossil fuel burning and land-use changes (Fig. 8). In the model, the carbon isotope signature of the atmosphere is updated for each year after a simple balance between carbon fluxes into and out of the atmosphere.




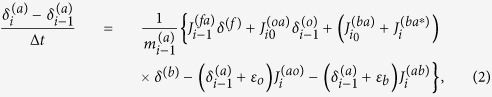




In equation (2) *J* denotes fluxes (Gt C/year), *m* denotes mass (Gt C), *t* denotes time (years), and superscripts *a, f, b*, and *o* denote the four carbon reservoirs: atmosphere, fossil fuel, terrestrial biosphere, and ocean mixed layer. The double superscripts denote the direction of the flux, i.e. ‘ao’ indicates the flux of carbon from the atmosphere to the ocean mixed layer. Subscript *i*_*0*_ indicates the initial time. The changes caused by uptake of carbon by the ocean and biosphere sinks were modified by adding *ε*_o_ and *ε*_b_ (units %). These two parameters correct for the global isotope fractionation between the atmosphere and the ocean and terrestrial biospheres. Because the isotope fractionation is affected by a range of processes and exact values are difficult to obtain, values were found by regression, fitting the initial (1860) modelled δ^13^C to the measured data. Since it is the carbon in the terrestrial biosphere rather than in the ocean mixed layer that dominates in biogenic uptake, we chose to use *ε*_*o*_ = 0.0%, and to estimate *ε*_*b*_ by a regression analysis giving a satisfactory steady state isotope value at the beginning of the forward model (i.e. 1860). Because of the interdependency of *ε*_*o*_ and *ε*_*b*_, other values of *ε*_*o*_ would lead to a different *ε*_*b*_, but modelled results after 1860 were found to not differ significantly. Values for the flux of carbon from burning of fossil fuel (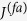) and the excess flux of carbon from changes in land use (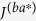) were obtained from Boden *et al.*^47^ and Houghton *et al.*^48^ respectively (Fig. 7). The remaining fluxes were estimated (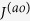 and 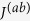) or assumed constant with time (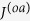 and 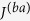). Out of the total (gross) fluxes of carbon between the atmosphere and biosphere, we used the Net Primary Productivity (NPP) to represent carbon fluxes that modify 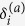. We assumed the NPP is about half of the Gross Primary Productivity (GPP), following Beer *et al.*^39^, and used an initial pre-industrial value for the GPP and NPP fluxes of 120 and 60 Gt/a respectively^5^. The 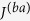 flux was then kept constant over the simulated time, with the model (Eq. 2) taking explicitly into account the changes in net fluxes from land-use changes.

(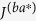) Fluxes from the atmosphere to the biosphere were updated with time, taking into account the CO_2_ fertilization effect on the biosphere following the increased levels of atmospheric CO_2_ (see Eq. 4 below). The carbon fluxes from the atmosphere to the ocean and terrestrial biosphere were estimated by assuming that a fraction of the excess carbon provided to the atmosphere from burning of fossil fuel and changes in land use has been accommodated by the ocean and terrestrial biosphere sinks:




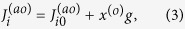




and




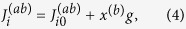




where




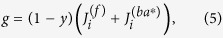




where *y* denotes the airborne fraction of CO_2_, i.e. the anthropogenic carbon being left in the atmosphere, and *x*^(*o*)^ and *x*^(*b*)^ the fractions of residual carbon being stored in the ocean and terrestrial biosphere sinks respectively (*x*^*(b)*^ *+ x*^*(o)*^ = *1*). A value of 0.46 was chosen for airborne fraction and assumed constant from 1860 to 2010, in accordance with estimates by e.g., Sabine *et al.*^49^, Knorr^50^, and IPCC^5^. The fractions of excess carbon being stored in the ocean and terrestrial biospheres are uncertain, and we therefore included a sensitivity study varying this fraction. Finally, the δ^13^C of fossil fuel emissions was taken from estimates by Andres *et al.*^34^, changing from about −24 in 1860 to about −28 at present.

Estimating the surface ocean δ^13^C and changes in this value with time is complicated by mixing of carbon from the atmosphere, terrestrial input, mixing of deep-ocean and surface-ocean water masses, and preferential removal of ^12^C over ^13^C by the oceanic biosphere. The sum of these processes leads to a positive δ^13^C. The effect of increasing atmosphere CO_2_ on the biogenic removal of carbon from the surface ocean water is not well understood and the surface ocean δ^13^C was therefore modified as a fraction *σ* of the change in the atmospheric δ^13^C:




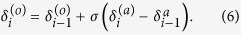




The exact value of *σ* is not known, but values close to 0.5 has been indicated^11^, and we used this value in the base case and then varied from 0.3 to 0.7 to illustrate the sensitivity of the model to this parameter. As will be demonstrated, using *σ* = 0.5 provides a drop in surface ocean δ^13^C of 0.95% that compares reasonably with other, more sophisticated, models that predict a drop of approximately 1% from 1800 to 1980^10^.

The mass of atmospheric carbon was updated with the assumption that the airborne fraction of CO_2_ was 46%, in accordance with estimates by e.g., Sabine *et al.*^49^. The corresponding changes in the CO_2_ pressure were estimated by:




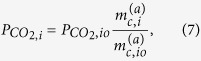




with an 1860 CO_2_ pressure value (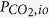) of 281 ppm.

## Additional Information

**How to cite this article**: Hellevang, H. and Aagaard, P. Constraints on natural global atmospheric CO_2_ fluxes from 1860 to 2010 using a simplified explicit forward model. *Sci. Rep.* **5**, 17352; doi: 10.1038/srep17352 (2015).

## Figures and Tables

**Figure 1:**
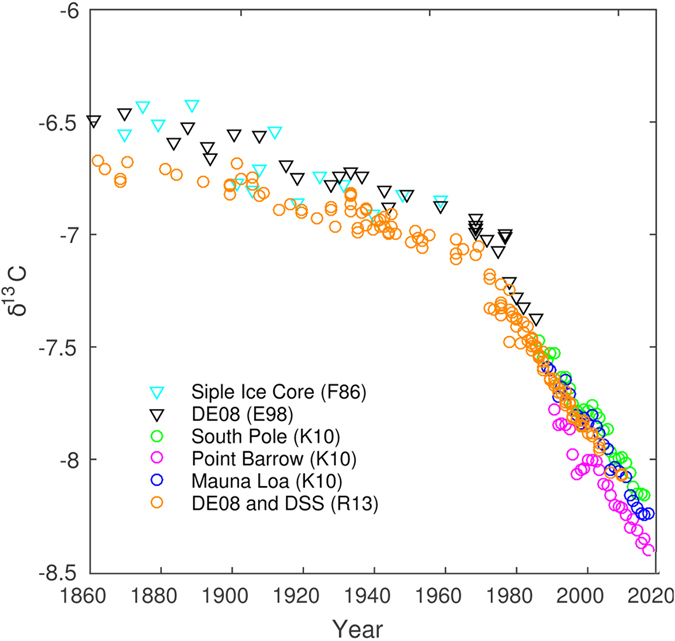
Measured stable carbon isotopes (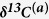) from 1860 to 2010. Data are from Friedli *et al.*^30^ (F86), Etheridge *et al.*^31^ (DE08, Law Dome Ice Core) and the recent series (South Pole, Point Barrow and Mauna Loa) from Keeling *et al.*^32^.

**Table 1:** Summary and explanation of input parameters used in the model.

Global carbon cycle parameters	Symbol used in model	Base-case value	References
Base terrestrial-atmosphere fluxes^*^	*J* ^*(ba)*^ *, J* ^*(ab)*^	60 Gt C/a (Half of the GPP)	5, 38
Base-case initial (1860) ocean-atmosphere fluxes	*J* ^*(oa)*^ *, J* ^*(ao)*^	78 Gt C/a	5
Fossil fuel fluxes	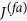	Time series, at present ~9 Gt C/a	48
Carbon fluxes from land-use changes	*J* ^(ba*)^	Time series, at present ~1.5 Gt C/a	49
Airborne fraction	*y*	46%	5, 50, 51
Relative contributions of the ocean and terrestrial biosphere sinks	*x*^(*o*)^*, x*^(*b*)^ *x*^(*o*)^ + *x*^(*b*)^ = *1*	50/50	Starting point for sensitivity
Factor relating change in ocean (mixed zone) δ^13^C relative to changes in the atmosphere δ^13^C.	*σ*	0.5	This model
Isotope composition of fossil fuel	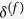	Time series, at present ~−28.0	30
Isotope composition of the terrestrial biosphere	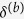	−25.0	5
Initial (1860) isotope composition of the ocean (mixed zone)	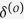	+2.5	
Initial (1860) isotope composition of the atmosphere	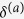	−6.48	
